# The AMEE-PPI Method to Extract Typical Outcrop Endmembers from GF-5 Hyperspectral Images

**DOI:** 10.3390/s25196143

**Published:** 2025-10-04

**Authors:** Lin Hu, Jiankai Hu, Shu Gan, Xiping Yuan, Yu Lu, Hailong Zhao, Guang Han

**Affiliations:** 1 Faculty of Land and Resources Engineering, Kunming University of Science and Technology, Kunming 650093, China; 18508770991@163.com (J.H.); yxp_kust@163.com (X.Y.); 20242101058@stu.kust.edu.cn (Y.L.); ghkmust@126.com (G.H.); 2 School of Geography and Planning, Sun Yat-sen University, Guangzhou 510006, China; zhaohlong6@mail2.sysu.edu.cn

**Keywords:** AMEE-PPI endmember extraction algorithm, GF-5, hyperspectral image, outcrop

## Abstract

**Highlights:**

AMEE-PPI combines the AMEE and PPI approaches for improved endmember extraction.

AMEE-PPI outperforms PPI, OSP, VCA, and AMEE in endmember extraction accuracy.

AMEE-PPI yields outcrop endmembers for geological exploration and spectral analysis.

**What are the main findings?**

- A hybrid algorithm named AMEE-PPI was proposed by integrating Automated Morphological Endmember Extraction (AMEE) and Pure Pixel Index (PPI), effectively overcoming limitations of each method and enhancing the precision and stability of endmember extraction from GF-5 hyperspectral images. The algorithm dynamically calculates pixel purity by running PPI within morphological structural elements, thus incorporating both spectral and spatial information.
- Experimental results on GF-5 hyperspectral images in a geologically complex outcrop region demonstrated that AMEE-PPI achieved superior performance compared to four classical algorithms (PPI, OSP, VCA, and AMEE), with the lowest Spectral Angle Distance (SAD) and Spectral Information Divergence (SID) values across all outcrop types. The extracted endmembers more closely matched ground-truth spectra, significantly improving hyperspectral representation of pure land-cover classes.

**What is the implication of the main finding?**

- The AMEE-PPI algorithm offers a more robust and accurate approach for endmember extraction in hyperspectral imagery, which is crucial for improving the quality of spectral unmixing, material classification, and object detection in remote sensing applications.
- By accurately identifying typical outcrop endmembers, the proposed method provides valuable spectral references for geological mapping, mineral exploration, and environmental monitoring, particularly in regions with complex surface compositions.

**Abstract:**

Mixed pixels remain a central obstacle to reliable endmember extraction from hyperspectral imagery. We present AMEE–PPI, a hybrid method that embeds the Pure Pixel Index (PPI) within morphological structuring elements and propagates spectral purity via dilation/erosion, thereby coupling spatial context with spectral cues while avoiding a user-fixed number of projections. On GaoFen-5 (GF-5) AHSI data from a geologically complex outcrop region, we benchmark AMEE–PPI against four widely used algorithms—PPI, OSP, VCA, and AMEE. The pipeline uses HySime for noise estimation and signal-subspace inference to set the endmember count prior to extraction and applies morphological elements spanning 3 × 3 to 15 × 15 to balance spatial support with local heterogeneity. Quantitatively, AMEE–PPI achieves the lowest spectral angle distance (SAD) for all outcrop types—purple–red: 0.135; yellow–brown: 0.316; gray: 0.191—surpassing the competing methods. It also attains the lowest spectral information divergence (SID)—purple–red: 0.028; yellow–brown: 0.184; gray: 0.055—confirming superior similarity to field reference spectra across materials. Visually, AMEE–PPI avoids the vegetation endmember leakage observed with several baselines on purple–red and gray outcrops, yielding cleaner, more representative endmembers. These results indicate that integrating spatial morphology with spectral purity improves robustness to illumination, mixing, and local variability in GF-5 imagery, with direct benefits for downstream unmixing, classification, and geological interpretation.

## 1. Introduction

Hyperspectral remote sensing technology is a ground object information recognition technology that combines target detection and spectral imaging, which changes the perspective of human observation of the earth’s surface with fine spectral resolution [1]. Hyperspectral images are widely used in many fields due to their narrow spectral band, which can provide a large amount of spectral information for each pixel [2]. However, due to the complexity of the distribution of ground objects and the limitation of the spatial resolution of remote sensing images by sensors, one pixel usually contains several different substances, which are called mixed pixels. The existence of mixed pixels reduces the accuracy of ground object recognition and becomes difficult, restricting the development of hyperspectral technology [3,4]_._ To improve the precision of remote sensing applications, the problem of mixed pixels must be solved. It is usually assumed that some pixels (known as endmembers) include only one ground object in their image, and the process of finding these endmembers is referred to as endmember extraction [5]. The extraction of appropriate endmembers is the key to pixel unmixing and can also be used in target detection, material identification, ground object classification, and other fields [6]. Therefore, the study of endmember extraction technology is of great significance.

Many researchers have extensively studied endmember extraction algorithms [7]. Bowles [8] proposed a method for understanding hyperspectral data using convex geometry, highlighting the convex, monolithic structure of hyperspectral data in multidimensional space. Building on this, Theile and Lavenier [9] introduced the Pure Pixel Index (PPI) algorithm, a standard endmember extraction technique. Other algorithms have also emerged, such as N-FINDR [10], Vertex Component Analysis (VCA) [11], and the Sequential Maximum Angle Convex Cone (SMACC) algorithm [12], each leveraging the spatial and geometric characteristics of the linear spectral mixing model. Plaza [13] introduced the Automated Morphological Endmember Extraction (AMEE) algorithm, which effectively incorporates spatial information. At the same time, numerous scholars have proposed improvements to these classical algorithms, enhancing their application to hyperspectral image processing. Liu [14] introduced the Maximum Volume by Householder Transformation (MVHT) method, overcoming the inconsistencies of VCA and the high computational cost of the Simplex Growing Algorithm (SGA) in calculating simplex volume. When compared to two hyperspectral datasets, MVHT outperformed both VCA and SGA. Nouri [15] used an optical real-time adaptive spectral identification system (ORASIS) to estimate endmembers across three datasets, finding it superior to VCA and PPI when evaluated with the Spectral Angle Mapper (SAM) and Spectral Feature Fitting (SFF) methods. Wang [6] proposed an endmember extraction model based on nonnegative tensor factorization (EIC-NTF), which mitigates the effects of high correlations between spectral features and abundance. This model was demonstrated to be effective for hyperspectral demixing through the extended multiplicative update framework. Liu [16] developed a Bayesian decomposition model that incorporates spectral variability, proving its efficacy in estimating endmembers and spectral variability in both synthetic and real datasets. Recent advancements in endmember extraction have seen the integration of deep learning (DL). Ozkan et al. [17] proposed an autoencoder-based method, EndNet, for endmember extraction and hyperspectral unmixing, outperforming existing techniques in various datasets. Shah et al. [18] introduced the Geo-Stat Endmember Extraction (GSEE) algorithm, which leverages statistical and geometric features of hyperspectral data. Simulation results indicate its strong performance. Hong et al. [19] developed the Endmember Guided Unmixing Network (EGU-Net), a DL-based method that considers endmember properties extracted from hyperspectral images. Tested on three datasets, EGU-Net demonstrated superior performance compared to state-of-the-art unmixing algorithms. Current endmember extraction techniques can be broadly categorized into algorithms based on traditional geometric theories (e.g., MVHT), new models (e.g., EIC-NTF, Bayesian decomposition), optical systems (e.g., ORASIS), deep learning (e.g., EndNet, EGU-Net), and statistical–geometric features (e.g., GSEE). The AMEE-PPI algorithm, as a classical method, continues to serve as a foundational reference for subsequent research. Together with these advanced techniques, AMEE addresses endmember extraction from diverse perspectives, driving the field forward. Each approach offers unique advantages and is suited to different application scenarios in hyperspectral image analysis. Beyond unmixing, recent AI-driven progress in hyperspectral anomaly detection (HAD) provides complementary insights that can benefit endmember extraction. In particular, Li et al. propose learning disentangled priors (LDP)—a coupling model-driven and data-driven framework that explicitly separates a physics-inspired low-rank background prior from learnable priors via deep networks and links them through residual connections—achieving state-of-the-art HAD performance across benchmarks [20]. While HAD and endmember extraction target different objectives, such physics-aware deep unfolding ideas (e.g., low-rank/sparse regularization and prior disentanglement) can be adapted to stabilize pure-pixel search under low SNR, spectral variability, and background interference, thus informing future DL-based unmixing designs.

Meanwhile, outcrops are part of rocks, veins, and ore deposits exposed to the ground, which provide direct evidence for the deposits and have significant scientific significance [21]. Traditional PPI- and morphology-based methods either treat the image as an unordered set of spectra and ignore spatial context (PPI) or rely on a morphological eccentricity index (MEI) whose reference changes across windows (AMEE), making purity scores incomparable across locations. Moreover, classical PPI requires an ad hoc choice of projection count (“skewer number”). To address these gaps, we propose AMEE-PPI, which computes local PPI within morphological structuring elements and accumulates purity across multi-scale dilation, unifying spatial and spectral cues; removes the MEI inconsistency by using a single, interpretable purity index (PPI) across windows; and obviates skewer tuning by generating projection vectors from pairwise connections among pixels within each window, so the number of projections follows window size automatically. The endmember extraction performance of the AMEE–PPI algorithm was evaluated and compared with that of PPI, orthogonal subspace projection (OSP), VCA, and AMEE. The AMEE–PPI algorithm was applied in GF-5 hyperspectral images to extract typical outcrop endmembers. The obtained typical outcrop endmember data provide primary data for further studies on hyperspectral technology and a theoretical basis and technical support for acquiring typical outcrop endmembers. The number of endmembers is an essential parameter of the endmember extraction algorithm, which affects the accuracy of endmember extraction in hyperspectral images. Hyperspectral Signal Subspace Identification by Minimum Error (Hysime) is a hyperspectral subspace identification algorithm proposed by José [22,23]. After estimating the correlation coefficient matrix of signal and noise by the HySime algorithm, the subspace with the minimum mean square error before and after projection is selected in the space composed of signal feature vectors, and the number of feature vectors constituting the subspace is the number of endmember estimations. The algorithm is an unsupervised, fully automatic (does not involve any parameters to be adjusted) feature decomposition algorithm based on minimum mean square error. This paper will use PIE-Hyp 1.0 software to implement the HySime algorithm to estimate the number of endmembers of hyperspectral image data.

There are current challenges in hyperspectral feature extraction. Beyond endmember extraction, hyperspectral feature extraction still faces open problems: spectral variability and nonlinear mixing across illumination, atmosphere, sensor, and scale, which challenge linear models and pure-pixel assumptions; label scarcity and domain shift across sensors, and regions, limiting the transferability of learned spectral–spatial features; high dimensionality and computational cost for deep spectral–spatial networks in large-area applications; noise and defective bands that necessitate robust denoising/band screening; and pipeline sensitivity to atmospheric/radiometric correction and spatial-scale settings, as well as limited interpretability/uncertainty quantification. These pain points motivate our hybrid spatial–spectral design and the preprocessing adopted for GF-5 AHSI in this work.

Therefore, this work pursues three objectives: (a) to develop AMEE–PPI, which runs PPI inside multi-scale morphological structuring elements and accumulates purity via dilation/erosion, thereby coupling spatial context with spectral purity and removing ad hoc skewer settings; (b) to determine the endmember number with HySime and rigorously benchmark AMEE–PPI against PPI, OSP, VCA, and AMEE on GF-5 AHSI outcrops using SAD and SID as quantitative criteria; and (c) to validate that AMEE–PPI yields cleaner, more representative outcrop endmembers than original hyperspectral averages with respect to ASD FieldSpec3 reference spectra, analyze typical failure modes (e.g., vegetation leakage), and discuss implications for unmixing, classification, and geological interpretation.

## 2. Materials and Methods

### 2.1. Study Area

**Figure 1:**
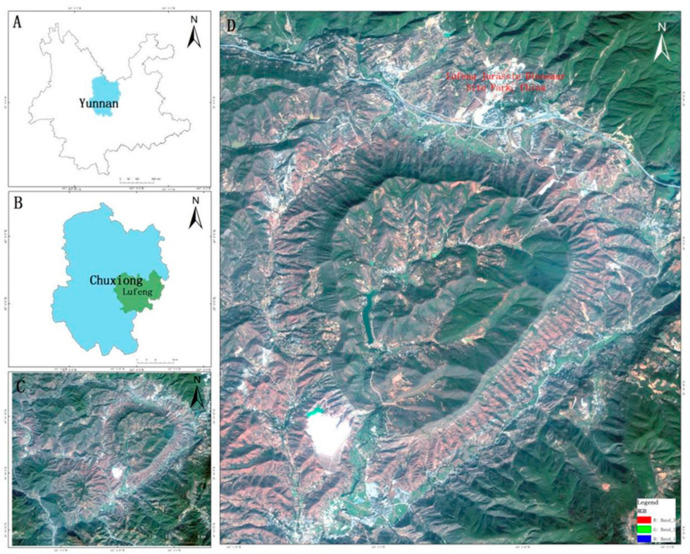
Study area (**A**) Yunnan Province; (**B**) Chuxiong City; (**C**) Dinosaur Valley Ring Structure Area; (**D**) Satellite Image of the Dinosaur Valley Ring Structure Area.

The study area is a typical mountainous ring structure (24°53′50″~24°59′21″ N, 102°06′30″~102°01′17″ E) at the southern edge of the Jurassic Dinosaur Ruins Park (World Dinosaur Valley) in Lufeng, Yunnan, China (As shown in Figure 1). The study area is a region of remarkable geographic diversity, which is manifested in several dimensions, such as topography and geomorphology, geology and tectonics, climate and hydrology, soil and vegetation, biodiversity, and cultural and human activities [24]. The topography of the region is complex and varied, showing unique geomorphological patterns such as craters and tectonic plateaus, and a rich variety of geologic types, including uplift and fracture tectonics, which have been formed by tectonic activities. The climate and hydrological characteristics are diverse, and the river system is well-developed, forming a variety of hydrological landscapes. The soil and vegetation types in the region show significant vertical zonal distributions owing to differences in rocks, topography, and climate. In terms of biodiversity, the region is rich in animal and plant resources and diverse ecosystem types, with a clear vertical ecosystem structure, while the diversity of culture and human activities is reflected in its rich historical heritage and multi-ethnic cultural landscapes. This comprehensive geographic diversity makes the Lufeng Dinosaur Valley Craton in Chuxiong an important geological research object and a key area for ecological protection and sustainable development.

### 2.2. Dataset Description and Data Processing

#### 2.2.1. GF-5 Outcrop Data and Preprocessing

GF-5 spaceborne hyperspectral data is archived by the GF-5 satellite in China and provided by the Yunnan Applied Research Center for Earth Observation Data. The GF-5 AHSI hyperspectral remote sensing satellite was successfully launched on 9 May 2018, and its basic parameters are shown in Table 1 [25,26]. This data source has the characteristics of high temporal resolution and high spectral resolution [26].

**Figure 2:**
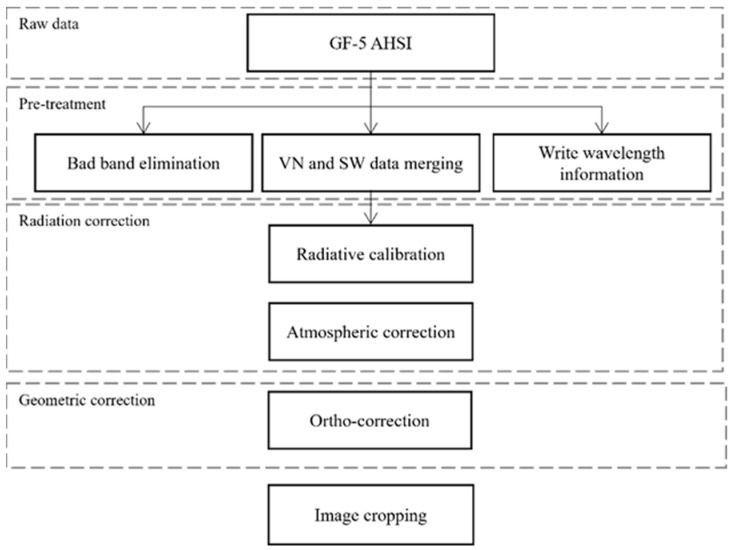
GF-5 preprocessing flowchart.

The study data used in this paper is a GF-5 satellite image acquired on 23 December 2019. According to the specific GF-5 satellite hyperspectral load parameters in Table 1, the GF-5 onboard hyperspectral data were preprocessed with bad band rejection, radiometric calibration, atmospheric correction, and orthometric correction [27] (the specific preprocessing flow is shown in Figure 2). Damaged and noisy bands (B151–154, B192–203, B246–267, B314–316, and B325–330) were removed from the images, and the remaining 283 bands were used in subsequent experiments.

The purple–red, yellow–brown, and gray outcrops in the annular structure region of Lufeng Dinosaur Valley, Chuxiong, Yunnan Province, as important components of geological exposures, reveal many unique characteristics of the area in terms of rock type, mineral composition, and geological evolution. The purple–red outcrops may be associated with iron oxides (such as hematite or wüstite), indicating the formation of rocks in a specific oxidative environment; the yellow–brown outcrops may reflect the presence of certain amounts of sulfides or iron oxide minerals in the rocks, with the distribution of these minerals being closely related to regional tectonic activity and surface chemical processes; while the gray outcrops likely represent the dominance of carbonate, silicate, or clay minerals in the rocks. The stability of these minerals corresponds to the unique geological background and evolutionary history of the region.

These outcrops of different colors not only reflect the complexity of the regional geological structure but also reveal the rock’s original environment, tectonic stress action, and subsequent modification processes. From the perspective of geographical diversity, their spatial distribution is closely related to the uplift, subsidence, and fault activity of annular structures, forming unique rock landforms and structural expressions. The distribution of purple–red and yellow–brown outcrops may be associated with tectonic thrust belts or fault systems, whereas gray outcrops are likely concentrated on relatively stable platforms or gentle hill terrains. Additionally, the color contrast of these outcrops enhances the visual effect of the regional geological landscape, providing an important natural resource for geological research and tourist observation. Furthermore, outcrops of different colors record the diversity of a region’s geological evolution, reflecting the differences in rock sources, interactions, and evolutionary processes.

**Figure 3:**
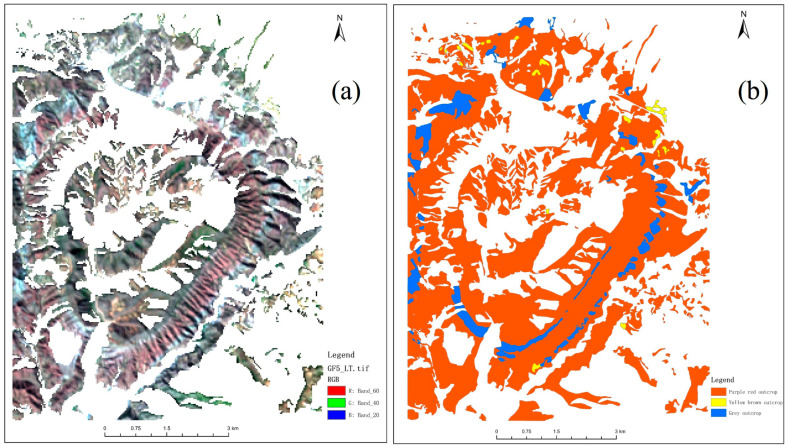
Outcrop in the study area, (**a**) outcrop dataset of GF-5 hyperspectral satellite images, (**b**) outcrop classification results.

Because the research object of this paper is the outcrop, in order to reduce the impact of other types of ground objects on the experimental results, we reference the same period of high spatial resolution remote sensing images through visual interpretation; the outcrop area in the study area is extracted, as shown in Figure 3a. According to Figure 3a, it can be seen that the outcrops are distributed in a zonal ring, mainly in the circular structure of the study area, and the rest are scattered in the north and southwest of the study area. Considering that hyperspectral remote sensing technology can realize the refinement and classification of outcrops in the study area, the outcrops in the study area are divided into three types by combining the remote sensing images with high spatial resolution, natural geography, and regional geological survey of the study area in the same period, and to the field exploration, the outcrops in the study area are divided into three kinds, namely purple–red outcrop, yellow–brown outcrop, and gray outcrop [28]. The classification results of outcrops are shown in Figure 3b. Then, the vector file of the outcrop classification results is used to cut the GF-5 satellite image for subsequent research.

#### 2.2.2. Standard Spectral Data of Ground Measurement

**Figure 4:**
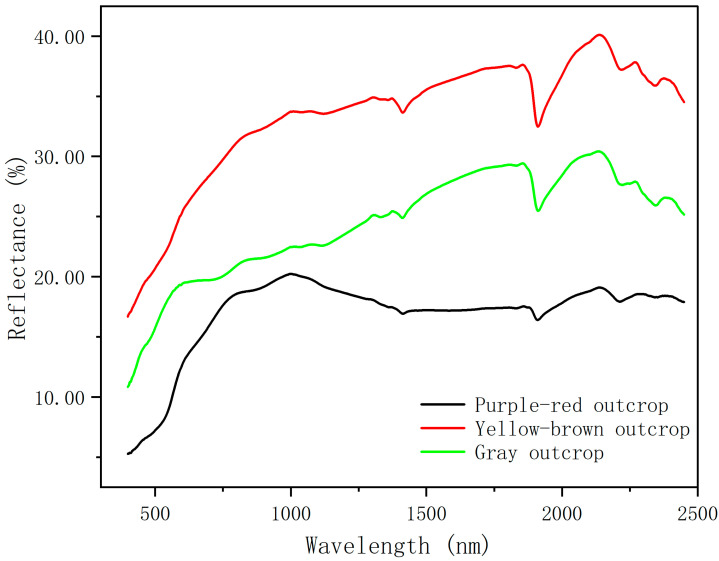
Standard spectral curves of three outcrops.

The endmember extraction experiment of GF-5 hyperspectral spaceborne data is based on the hyperspectral data of field outcrop samples taken by the ASD Field Spec3 ground object spectrometer as the standard data. One soil sample from each of the three types of outcrops was collected in the field, and the hyperspectral data of the three types of outcrops were measured using an ASD FieldSpec3 ground spectrometer after grinding and sieving five times in the laboratory. The spectral curves from the five measurements were averaged and then smoothed by the SG to obtain the ground-truth standard spectral curves for the three types of outcrops, as shown in Figure 4.

### 2.3. Methodology

#### 2.3.1. Endmember Extraction Comparison Algorithms

##### PPI

**Figure 5:**
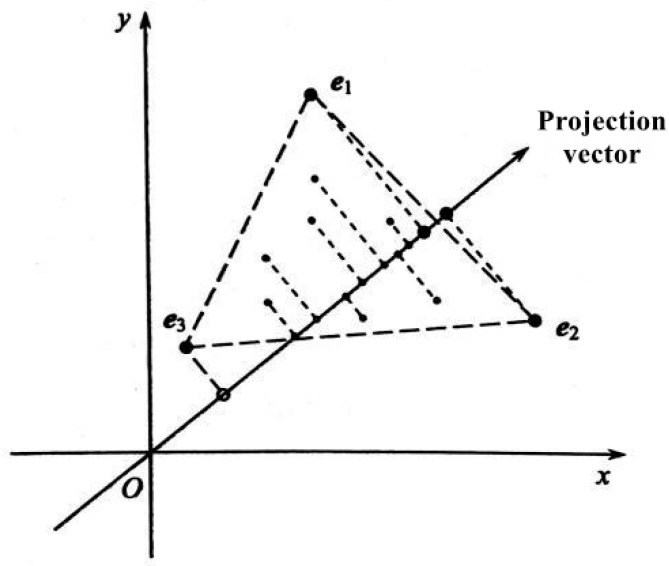
Principle of PPI algorithm OSP.

The PPI algorithm is based on the geometric description of the linear mixed model. The endmembers are extracted by using the features of the endmembers, which are the vertices of the simplex formed in the feature space of hyperspectral remote sensing images and the vector projection property of the simplex [29,30,31]. Figure 5 shows the basic principle of the PPI algorithm, and $e_{1}$, $e_{2}$, and $e_{3}$ are three data points obtained by the PPI algorithm, shown in the figure. In the eigenspace, if you take the inner product of any vector with a unit vector, you will obtain the number of projection vectors in this unit vector direction. The same direction is positive, the reverse is negative, and the orthogonal is 0. If the exponent of the pure pixel corresponding to a pixel is larger, it means that the pixel is more likely to be an endmember.

##### OSP

The algorithm is based on the geometric description of the linear spectral mixture model. Firstly, a region of interest (ROI) is constructed, and the region perpendicular to it is a non-interest region (NROI) [32]. Then, a vector is generated at the vertex pixel of a simplex composed of pixels. All vectors are projected to the ROI, and the endmembers are identified based on the distance of the projection. OSP algorithm can shield the information of the non-interest direction to the maximum extent so that the information of the ROI can be well-preserved [33]. However, the construction of ROI and NROI is the difficulty of this algorithm. The advantage of orthogonal subspace projection is that it can avoid the repeated extraction of endmembers to the maximum extent, but the experimental results are susceptible to image noise.

##### VCA

Based on the geometric description of the linear spectral mixture model, VCA extracts endmembers one by one by repeatedly searching for orthogonal vectors and calculating the projection distance of the image matrix on the orthogonal vectors [34]. Several vertices of a simplex can be stretched into a subspace, and the maximum point of the projection distance of a simplex on a vector orthogonal to this subspace must be the vertex of the simplex.

##### AMEE

AMEE algorithm not only uses spectral features but also introduces spatial information into the endmember determination process. AMEE algorithm expands the spectral dimension by comparing dilation and erosion operations of image morphology and uses the cumulative distance measure to measure the grade of pixels, thus defining the morphological operations on the spectrum.

Assuming that any image coordinate (x,y) is a pixel f(x,y), a core range K(x,y) is defined. Generally, this core range is selected as a square with center (x,y) and side length 2k+1, and the value of the core range width k is given according to the actual situation. In K(x,y) of any two pixels $f(x_{1},y_{1})$, $f(x_{2},y_{2})$, there is a distance $dist[f(x_{1},y_{1}),f(x_{2},y_{2})]$ in the feature space, which can be Euclidean distance or spectral angular distance. Then, the cumulative distance of a pixel $f(x^{\prime },y^{\prime })$ within K(x,y) is

(1) $$\begin{array}{c} D\left[f\left(x^{\prime },y^{\prime }\right),K\left(x,y\right)\right]=\underset{\delta }{\sum }\underset{t}{\sum }dist\left[f\left(x^{\prime },y^{\prime }\right),f\left(s,t\right)\right] \end{array}$$

Therefore, dilation and erosion operations in hyperspectral images are defined as follows:

(2) $$\begin{array}{c} d(x,y)=\arg[\underset{\left(\delta ,t\right)\in k\left(x,y\right)}{max}\left\{D\left[f\left(s,t\right),K\left(x,y\right)\right]\right\}] \end{array}$$

(3) $$\begin{array}{c} e(x,y)=\arg[\underset{\left(\delta ,t\right)\in k\left(x,y\right)}{min}\left\{D\left[f\left(s,t\right),K\left(x,y\right)\right]\right\}] \end{array}$$

where d(x,y) is the dilation operation and e(x,y) is the erosion operation. Note that d(x,y) and e(x,y) are image coordinates and not spectrum.

AMEE defines two endmember extraction processes, voting and evaluation, by using extended dilation and erosion operations. The voting process is to define the number of votes V(x,y) for each pixel (x,y) after the width k of the core area K(x,y) is given in advance. Then, each pixel (x,y) in the image is expanded in the core area K(x,y) to obtain a dilation with the largest cumulative distance d(x,y), and then the pixel d(x,y) obtains one vote, namely V(x,y) increase. When all pixels are dilated, namely, the voting is completed, the endmember can be judged according to the number of votes received by each pixel.

The evaluation process is an improvement of the voting process. After the width k of the core area is given in advance, one morphological eccentricity index (MEI) is defined for each pixel (x,y), denoted as MEI(x,y). Then, each pixel (x,y) in the image is dilated and eroded in the core region K(x,y) to obtain d(x,y) and e(x,y). The morphological eccentricity index MEI(x,y) of the pixel d(x,y) increases $disk\left\{f[d(x,y)],f[e(x,y)]\right\}$. After all pixels are dilated and eroded, the endmember can be determined according to the MEI of each pixel. If MEI is greater than a certain threshold, it is a pure pixel.

##### AMEE-PPI Algorithm

From the above introduction, we can see that the AMEE algorithm has some shortcomings. The AMEE algorithm defines the spectral angular distance between the purest pixel and the pixel with the maximum mixing degree as MEI and uses MEI to represent the purity of the pixel in the structural element. However, because the pixels with the largest mixing degree in different structural elements are different, the reference standards for MEI calculation are also different. Therefore, the size of MEI cannot effectively represent the purity of pixels. In addition, from the perspective of the existing PPI endmember extraction algorithm, the projection vector of the PPI algorithm is randomly generated each time, and the accuracy of obtaining endmembers cannot be guaranteed. The endmembers obtained by running the PPI algorithm multiple times in a graph may be different [35,36]. This method only regards the hyperspectral image as a set of disordered spectral vectors, only uses the spectral information of the image, and ignores the spatial structure information of the distribution of ground objects in the image.

**Figure 6:**
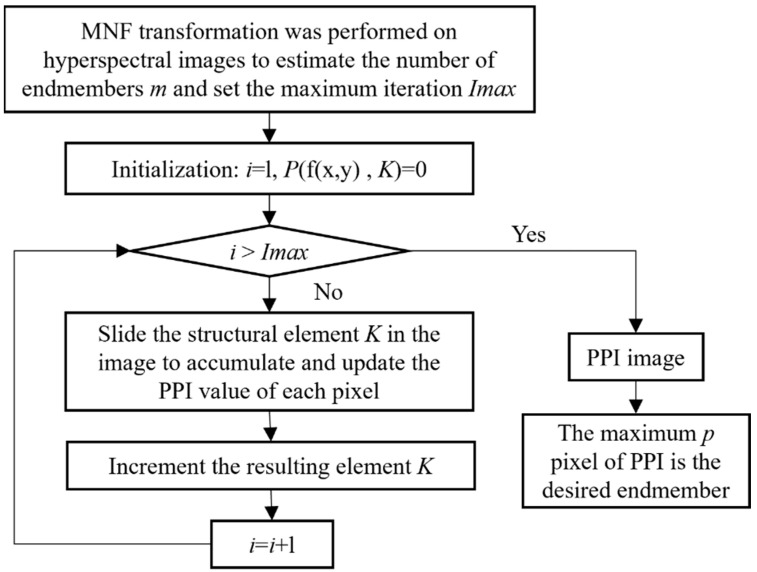
AMEE-PPI algorithm principal flowchart.

Therefore, inspired by the paper, the AMEE-PPI algorithm process [29] is shown in Figure 6. Consider replacing the MEI with the PPI to represent the purity of pixels in structural elements. The PPI algorithm is used to find the purest pixel in the structure elements, the corresponding purity index is assigned to the pixel, and the dilation and erosion operation in morphology is defined to process the whole image. The size of structural elements is constantly increased, and the PPI values of pixels are repeatedly calculated and updated cumulatively. Finally, an image with a PPI value can be obtained, and the endmember can be selected from the pixel with a larger PPI cumulative value. In this way, the shortcomings of the AMEE algorithm are remedied, and the performance of the AMEE algorithm is improved by combining the AMEE algorithm with the PPI algorithm, namely the AMEE-PI algorithm.

In this algorithm, the PPI algorithm runs inside the structure elements, and the pixels of the structure elements are connected in the feature space in pairs to generate projection vectors. This allows projection vectors to be distributed as far as possible in different directions rather than in the traditional PPI algorithm. Because the generated projection vector is random, it may make the generated projection vector mainly focus on some specific directions, which makes the result inaccurate. Since the number of pixels contained in structural elements is determined, the number of projection vectors generated by pairwise lines is determined. With the dilation of structural elements, the number of pixels increases, and the generated projection vectors also increase automatically. It is no longer necessary for users to set the number of projection vectors subjectively, which improves the automaticity of the algorithm to a certain extent.

The specific steps of the AMEE-PPI algorithm are as follows:

(1) The image reduction and denoising process is realized by applying the minimum noise separation transformation to the whole image and estimating the number of end elements m.
(2) The minimum size K_min and maximum size K_max of the structural elements are set to derive the maximum number of iterations I_max.
(3) Make i = 1, the initial PPI value for all image elements P(f(x,y), K) = 0, and start the execution from the smallest structure element K_min.
(4) Expansion according to the morphological structure operator defined by the PPI algorithm expansion to obtain the purity index of each image element within the structure element.
(5) i = i + 1. If i = I_max, sequentially execute step (6); otherwise, increase the structure element K and return to step (4).
(6) The PPI image is output concerning m image elements with larger PPI values identified as end elements.

There are key differences from existing methods. Compared with classical PPI, VCA, and OSP that treat the hyperspectral image as a bag of spectral vectors, AMEE–PPI runs PPI inside growing morphological structuring elements and then propagates purity by dilation/erosion, thus integrating spatial structure with spectral cues. In contrast to PPI that requires a user-set number of projection vectors, AMEE–PPI generates pairwise projection vectors among pixels within each structuring element; the number of projections automatically increases with the element size.

### 2.4. Evaluation Index of Experimental Results

To evaluate the credibility of endmember extraction results is to determine whether the extracted endmember can truly represent a specific type of ground object. At present, spectral angle divergence (SAD) and spectral information divergence (SID) are widely used in the international community for similar measurement. If the obtained SAD and SID are smaller, it means that the extracted results are closer to the real image, and vice versa.

(1) SAD

(4) $$\begin{array}{c} SAD[e_{k},{\hat{e}}_{k}]=\arccos\left(\frac{\left[e_{k},{\hat{e}}_{k}\right]}{\left\|{\left.e_{k}\right\|}_{2}\cdot \left\|{\left.{\hat{e}}_{k}\right\|}_{2}\right.\right.}\right) \end{array}$$

where $e_{k}={\left\{e_{s}\right\}}_{S=1}^{L}$ represents the real endmember in the spectral library, ${\left\{{\hat{e}}_{s}\right\}}_{S=1}^{L}$ represents the extracted endmember, and L represents the number of bands.

SAD ∈ [0,π]; smaller is better. SAD measures shape similarity (it is gain/scale-invariant): 0 means identical direction (highest similarity); values < 1 indicate close matches; larger values indicate increasing angular mismatch. All SAD values reported in Table 2 and Table 3 are computed as extracted endmember vs. standard outcrop spectrum.

(2) SID

Let $e_{k}$ and ${\hat{e}}_{k}$ be two high-dimensional vectors, whose self-information is $I(e_{k})$ and $I({\hat{e}}_{k})$ respectively.

(5) $$\begin{array}{c} \left\{\begin{array}{c} I_{s}(e_{k})=-\log p_{s}\\ I_{s}(e_{k})=-\log q_{s} \end{array}\right. \end{array}$$

(6) $$\begin{array}{c} p_{s}=\frac{e_{s}}{\sum_{s=1}^{L}e_{s}},q_{s}=\frac{{\hat{e}}_{s}}{\sum_{s=1}^{L}{\hat{e}}_{s}} \end{array}$$

Here, $e_{k}={\left\{e_{s}\right\}}_{S=1}^{L}$ represents the real endmember in the spectral library, ${\hat{e}}_{k}={\left\{{\hat{e}}_{s}\right\}}_{S=1}^{L}$ represents the extracted endmember, and L represents the number of bands. The correlation entropy of the two vectors is $e_{k}$ and ${\hat{e}}_{k}$.

(7) D(ek||e^k)=∑s=1Lpslog2(psqs)

In the same way,

(8) D(e^k||ek)=∑s=1lqslog2(qsps)

Then the SID of $e_{k}$ and ${\hat{e}}_{k}$ is

(9) $$\begin{array}{c} SID\left(e_{k},{\hat{e}}_{k}\right)=D\left(e_{k}\left\|{\hat{e}}_{k}\right.\right)+D\left({\hat{e}}_{k}\left\|e_{k}\right.\right)\\ =\overset{L}{\underset{s=1}{\sum }}p_{s}\log_{2}(\frac{p_{s}}{q_{s}})+\overset{L}{\underset{s=1}{\sum }}q_{s}\log_{2}(\frac{q_{s}}{p_{s}}) \end{array}$$

SID ≥ 0 (unbounded above); smaller is better. SID compares the relative band-wise energy distributions; SID = 0 iff the normalized spectra are identical. Numerically stable computation uses a small ε before normalization to avoid log 0. All SID values in Table 2 and Table 3 are likewise computed between extracted endmembers and the standard outcrop spectra.

## 3. Results

### 3.1. Evaluation of Endmember Extraction Algorithms for Hyperspectral Images

**Figure 7:**
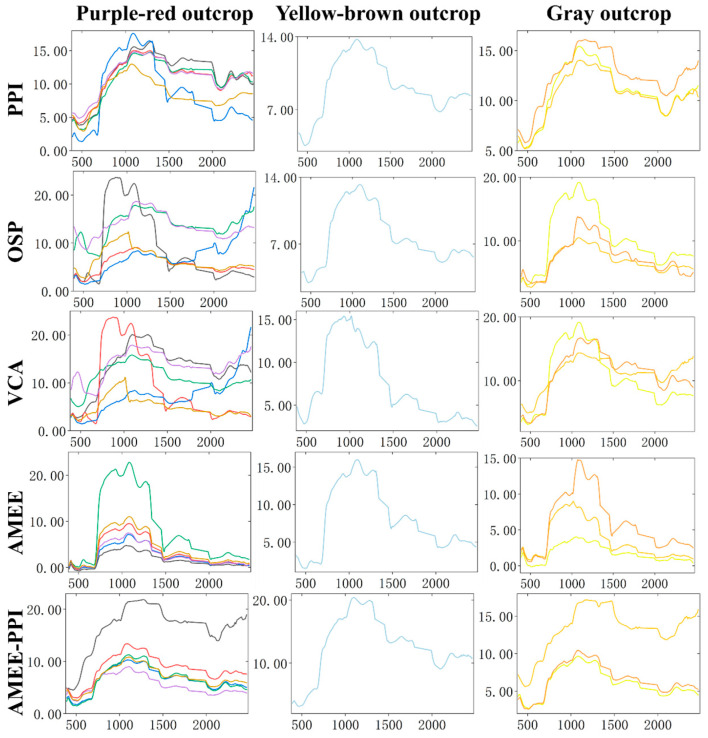
The endmember extraction results of GaoFen-5 (GF-5) hyperspectral spaceborne data. PPI: Pure Pixel Index, OSP: orthogonal subspace projection, VCA: vertex component analysis, AMEE: Automated Morphological Endmember Extraction.

The AMEE-PPI algorithm and four comparison algorithms (PPI, OSP, VCA, and AMEE) were used to perform endmember extraction experiments on regional images of different outcrops in the study area, and the endmember extraction results of outcrops were obtained as shown in Figure 7. Table 2 and Table 3 use SAD and SID as two indexes to evaluate the extracted outcrop endmembers and obtain the statistical tables of SAD and SID between the outcrop endmember spectrum extracted by various algorithms and the standard spectrum, in which the best performance is displayed in bold. In this experiment, it should be noted that (1) after noise estimation and signal-subspace inference of image data by the HySime algorithm, it is shown that the purple–red outcrop has 6 endmembers, yellow–brown outcrop has 1 endmember, and gray outcrop has 3 endmembers, which are used as the number setting standard of five endmember extraction algorithms; and (2) the minimum and maximum structural elements of AMEE and AMEE-PPI algorithms are set as 3 × 3 and 15 × 15, respectively.

Figure 7 shows that PPI, OSP, VCA, and AMEE all have the problem of extracting obvious vegetation endmembers when extracting purple–red outcrop and gray outcrop endmembers. The AMEE-PPI algorithm has no such problem. As can be seen from Table 2, the experimental results of GF-5 hyperspectral spaceborne data with AHSI as the sensor show that the minimum SAD value of the purple–red outcrop is 0.135, and that of the yellow–brown outcrop is 0.316. The minimum SAD value of the gray outcrop is 0.191. It can be seen that, compared with PPI, OSP, VCA and AMEE, the SAD value between the endmember extracted by the AMEE-PPI algorithm and the ground-measured standard spectrum is the smallest. The results in Table 3 show that the minimum SID value of the purple–red outcrop is 0.028, that of the yellow–brown outcrop is 0.184, and that of the gray outcrop is 0.055. It can be seen that the SID value between the endmember extracted by the AMEE-PPI algorithm and the ground-measured standard spectrum is also the smallest. By comparing the experimental results of the AMEE and AMEE-PPI algorithms, it can also be found that the SAD value of the AMEE-PPI algorithm is significantly reduced compared with that of the AMEE algorithm, and so is SID.

**Figure 8:**
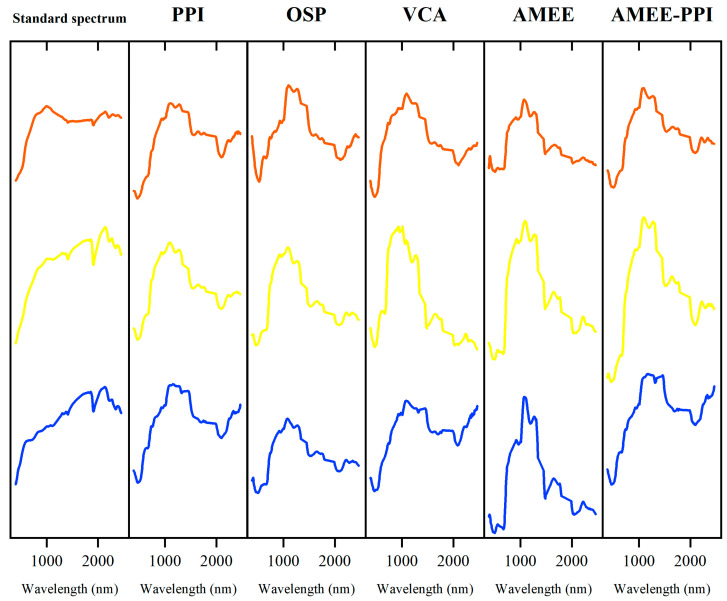
The spectral curves of the outcrop endmembers extracted by various algorithms. PPI: Pure Pixel Index, OSP: orthogonal subspace projection, VCA: vertex component analysis, AMEE: Automated Morphological Endmember Extraction.

The endmember spectral curves of the purple–red outcrop, yellow–brown outcrop, and gray outcrop in the study area extracted by PPI, VCA, OSP, AMEE, and AMEE-PPI algorithm are shown in Figure 8 and compared with the ground-measured standard spectral curves. It can be seen from the experimental results that, compared with PPI, OSP, VCA, and AMEE, the endmember extracted by the AMEE-PPI algorithm has a higher similarity with the standard spectral curve.

### 3.2. Comparative Analysis of Outcrop Endmember and Original Hyperspectral

Through comparative analysis of outcrop endmembers and original hyperspectral, the effectiveness of endmember extraction processing in obtaining hyperspectral curves of pure ground objects in images is verified. Taking SAD and SID as evaluation indexes and ground-measured spectrum as a standard spectrum, the outcrop endmember and original spectrum were quantitatively evaluated to realize the comparative analysis of outcrop endmember and original hyperspectral. Hyperspectral curves of three outcrop endmembers in the study area extracted by the AMEE-PPI algorithm were used as the outcrop endmember data. Taking the vector file obtained in Figure 3b as the ROI, the average spectral curves of the three outcrops were calculated respectively. The average spectral curves were the original hyperspectral data. The results shown in Table 4 are obtained through quantitative evaluation.

It can be found from Table 4 that the SAD and SID values of the outcrop endmember extracted by the AMEE-PPI algorithm are smaller than those of the original outcrop hyperspectral data. Among them, the SAD and SID values of the purple–red outcrop changed most significantly, which decreased by 0.077 and 0.068, respectively. However, the SAD and SID values of the yellow–brown outcrop had the least significant change, which decreased by 0.039 and 0.014, respectively.

## 4. Discussion

### 4.1. Comparison with Classical Algorithms and Method Characteristics

The AMEE-PPI algorithm combines the design ideas of the AMEE algorithm and the PPI algorithm. The PPI algorithm is used to define the dilation and erosion operations in mathematical morphology, and the purest pixel is found by accumulating the PPI of each pixel in the structure element. The PPI algorithm only takes the whole hyperspectral image as a disorder vector for projection and only uses the spectral information of the image. The AMEE-PPI algorithm runs the PPI algorithm in the structure element and then combines the dilation operation of mathematical morphology to process the whole image, taking into account the spatial information and spectral information of the image. The experimental results show that the endmember extraction accuracy of the AMEE-PPI algorithm is better than the PPI, OSP, VCA, and AMEE algorithms.

At the same time, it can also be found in the experimental process that the PPI algorithm uses the idea of convex geometry analysis to quickly extract endmembers by simply locating the purest pixel [29]. It has the advantages of simple operation, but there are also the disadvantages that the category selection needs prior knowledge and is sensitive to noise. The OSP algorithm and VCA algorithm are classical algorithms for endmember extraction based on convex geometric models, both of which are based on the assumption of pure pixels [37] (that is, there are pure pixels in the image). If there are no pure pixels in the image or a small number of pure pixels, the accuracy of the results will decrease. The OSP algorithm can improve the accuracy of spectral matching by using spatial information, but it is still inferior to the AMEE-PPI algorithm. The AMEE algorithm extracts endmembers based on the idea that the spectral angular distance between the purest pixel and the pixel with the maximum mixing degree is defined as the MEI5. This algorithm is superior to the extraction effect of only considering the spectral information, which can reasonably reflect the change in ground object content and avoid the large difference in abundance value between different pixels. However, the algorithm also has the disadvantages of high computational complexity and slow running speed.

Although the AMEE-PPI algorithm shows good performance in endmember extraction, it still has some limitations in practical applications. For example, AMEE-PPI is sensitive to noise and outliers, especially in low signal-to-noise ratio scenarios, which may lead to inaccurate extracted endmembers. At this time, a statistical analysis method with stronger noise robustness can be added to the PPI screening process for optimization. The AMEE algorithm relies on structural elements (such as window size) in morphological operations. The selection of different scales has a great influence on the results. An adaptive scale selection strategy can be introduced to dynamically adjust the size of the structural element according to local spatial features. In addition, both AMEE and PPI algorithms are relatively traditional methods. You can try to introduce deep learning models (such as autoencoders or convolutional neural networks) for preliminary extraction of endmembers and then use PPI for further screening.

### 4.2. Robustness Analysis and Future Work

Our hybrid design improves robustness in three ways. (i) Spatial–spectral integration: By running PPI within morphological structuring elements and propagating purity by dilation/erosion, AMEE–PPI leverages local spatial context to stabilize endmember selection under illumination and mixing variability compared with purely spectral approaches. (ii) Noise-aware subspace setting: We use HySime’s noise estimation and signal-subspace inference to determine the number of endmembers, reducing sensitivity to noisy dimensions when SNR is low. (iii) Noise-reduced reference and quantitative checks: Ground reference spectra are SG-smoothed, and SAD/SID to these references provide noise-aware, comparable accuracy metrics. Nevertheless, the method can be affected by very low SNR, scarce pure pixels, and the choice of structuring element scale. We therefore consider modern HSI denoisers and uncertainty-aware band screening, as well as variability-aware/nonlinear priors, as promising directions for further robustness.

Spectral variability and nonlinearity remain primary obstacles; jointly modeling endmember variability with spatial context improves robustness but does not fully remove the dependence on pure pixels in complex scenes. Future work will embed variability-aware and nonlinear priors (e.g., generative or attention-based unmixing) into our pipeline. Second, to address label scarcity and domain shift, we will explore few-shot/meta-transfer strategies and cross-sensor adaptation for GF-5 and airborne data. Third, computational efficiency is crucial for large-area mapping; lightweight spectral–spatial architectures and pruning/quantization will be evaluated to reduce inference cost while preserving accuracy. Finally, noise/bad-band robustness will be enhanced via modern HSI denoisers and uncertainty-aware screening so that downstream features remain stable when SNR is low.

In future research we will (i) embed variability-aware and nonlinear priors—e.g., generative or attention-based unmixing—to relax pure-pixel and linear-mixing assumptions; (ii) mitigate label scarcity and domain shift by few-shot/meta-transfer strategies and cross-sensor adaptation between GF-5 and airborne/field datasets; (iii) improve large-area mapping efficiency through lightweight spectral–spatial architectures coupled with pruning/quantization; (iv) increase robustness to noise and defective bands via modern hyperspectral denoisers and uncertainty-aware band screening; and (v) design an adaptive selection of morphological scales and evaluate deep networks as pre-extractors coupled with PPI screening.

## 5. Conclusions

This paper uses the AMEE-PPI algorithm to compare the four classic algorithms PPI, OSP, VCA, and AMEE, conducting simulation experiments on the GF-5 satellite hyperspectral dataset. The results show that, based on quantitative indicators (SAD value and SID value), the experiments using the GF-5 hyperspectral dataset indicate that the endmember extraction accuracy of the AMEE-PPI algorithm is generally superior to the other four algorithms. Moreover, the AMEE-PPI algorithm shows improved accuracy compared to the AMEE algorithm. Through qualitative image comparison, the AMEE-PPI algorithm generally outperforms the other four algorithms in terms of accuracy when extracting endmembers from different outcrops. In addition, the hyperspectral curves obtained after endmember extraction using the AMEE-PPI algorithm eliminate a large number of impurities, allowing for a more accurate representation of the hyperspectral characteristics of the ground objects compared to the original hyperspectral data.

However, experimental results also show that when there are fewer pure pixels in the image or the image is more discrete, the endmember accuracy obtained will decline. In future work, we will prioritize: (i) variability-aware/nonlinear unmixing, (ii) few-shot/meta-transfer and cross-sensor adaptation, (iii) efficient lightweight implementations, (iv) robustness via denoising and uncertainty-aware band screening, (v) adaptive morphological scales with deep pre-extractors plus PPI screening, and (vi) multi-source fusion and pipeline automation for scalable deployment.

## Figures and Tables

**Table 1:** Basic parameters of GF-5 satellite hyperspectral load.

**Spectral Range/nm**	**Spatial Resolution/m**	**Width/km**	**Spectral Resolution/nm**	**Number of Bands**	**Average Orbital Height/km**
400~2500	30	60	VNIR:5; SWIR:10	330	705

**Table 2:** SAD between the outcrop endmember spectrum extracted by various algorithms and the standard spectrum.

Outcrop	PPI	OSP	VCA	AMEE	AMEE-PPI
Purple–red outcrop	0.136	0.188	0.158	0.608	**0.135**
Yellow–brown outcrop	0.366	0.356	0.520	0.482	**0.316**
Gray outcrop	0.204	0.330	0.202	0.610	**0.191**

**Table 3:** SID between the outcrop endmember spectrum extracted by various algorithms and the standard spectrum.

Outcrop	PPI	OSP	VCA	AMEE	AMEE-PPI
Purple–red outcrop	0.030	0.066	0.040	1.002	**0.028**
Yellow–brown outcrop	0.195	0.197	0.486	0.461	**0.184**
Gray outcrop	0.060	0.168	0.062	0.788	**0.055**

**Table 4:** Comparison of outcrop endmembers and original hyperspectral results.

	Original Hyperspectral		Outcrop Endmember	
	SAD/Rad	SID/Bit	SAD/Rad	SID/Bit
Purple–red outcrop	0.211	0.096	0.135	0.028
Yellow–brown outcrop	0.355	0.199	0.316	0.184
Gray outcrop	0.257	0.095	0.191	0.055

## Data Availability

The GF-5 hyperspectral data that support the findings of this study are conditionally available from Yunnan Applied Research Center for Earth Observation Data. The data are not publicly available due to their containing information that could compromise the privacy of Yunnan Applied Research Center for Earth Observation Data.
